# A cross-country core strategy comparison in China, Japan, Singapore and South Korea during the early COVID-19 pandemic

**DOI:** 10.1186/s12992-021-00672-w

**Published:** 2021-02-22

**Authors:** Haiqian Chen, Leiyu Shi, Yuyao Zhang, Xiaohan Wang, Gang Sun

**Affiliations:** 1 Department of Health Management, School of Health Management, Southern Medical University, Guangzhou, Guangdong 510515 PR China; 2 Department of Health Policy and Management, Bloomberg School of Public Health, Johns Hopkins University, Baltimore, MD 21205 USA

**Keywords:** COVID-19, Containment strategy, Mitigation strategy, Core strategy comparison

## Abstract

**Background:**

The study aimed to analyze the effectiveness of COVID-19 strategies adopted by China, Japan, Singapore, and South Korea.

**Methods:**

We extracted publicly available data from various official websites, summarized the strategies implemented in these four countries, and assessed the effectiveness of the prevention and control measures adopted by these countries.

**Results:**

As of October 28, 2020, the growth of daily new confirmed cases has stabilized in China, Singapore, and South Korea. In Japan, the daily new confirmed cases increased sharply since it lifted a state of emergency, but case-fatality maintains at a lower level. The growth of total cases is near stagnant in China and Singapore, with a case-fatality of 5.39 and 0.05% respectively. The case-fatality rate between Japan and South Korea is identical at 1.76%, however, Japan’s growth rate of cases has increased more rapidly than South Korea.

**Conclusion:**

This study found that China, Japan, Singapore, and South Korea accessed the situation within their own borders and implemented different intervention strategies to curb the spread of COVID-19 and maintain lower rates of case-fatality. China, Singapore, and South Korea adopted the containment strategy, while Japan adopted the mitigation strategy. Although Japan’s case-fatality maintained at a low level, daily new cases increased faster than the other three countries. This result indicated that a mitigation strategy could be inferior to a containment strategy.

## Background

The Coronavirus Disease 2019 (COVID-19) pandemic is ongoing globally, which has severely impacted politics, economics, and culture. The first COVID-19 case was detected in Wuhan, China in December 2019. Then eventually spreading to other Asian nations and the rest of the world. It was found that COVID-19 spreads more rapidly compared to the Severe Acute Respiratory Syndrome (SARS) and the Middle East Respiratory Syndrome (MERS) [1]. As of Oct 28, 2020, it has affected 217 countries and territories around the world, causing 45 million infections and over 1.1 million deaths [2]. To control the global pandemic, governments around the world have adopted different intervention strategies (such as social distancing, quarantine, isolation, lockdown, curfews, travel restrictions, schools/colleges closing) to contain the spread of COVID-19 [3].

Based on the different countries have different concepts on the feasibility of blocking the virus transmission, estimation of disease severity, the social and economic effects of strategy, the acceptance and willingness of public, and the government willpower and capacity. Some countries adopted a strict containment strategy, which core interventions are proactive in detecting and managing cases, tracing and isolating close contacts, and strictly restricting or controlling population movements when feasible and appropriate [4]. China, Japan, Singapore, and Thailand are the representative countries for applying these measures. Whereas other countries implemented a relaxed mitigation strategy, which core measures are focus on the treatment of severe cases and utilizing non-pharmaceutical interventions, rather than optimizing the detection and management of each case and close contacts [5]. The typical representative country is Japan, which is located in East Asia. Of course also some European or Americas countries such as the United Kingdom, Italy, France, and the United States.

In this paper, we selected China, Singapore, and South Korea of East Asia. These countries implemented a strict containment strategy response to the earlier outbreak of COVID-19, and the epidemic was well controlled, even blocking the virus transmission successfully in the local area. Besides, we chose Japan as the representative country through adopting a mitigation strategy to control the epidemic, and the COVID-19 cases were not increased rapidly like other countries of Europe. These four countries have been successful in containing the spread of the COVID-19 outbreak by implementing useful containment or mitigation strategies. Focusing on these aspects, our study aimed to analyze the effectiveness of the intervention strategies adopted by China, Japan, Singapore, and South Korea. We hope this comparative analysis may be helpful and guide other countries towards developing an effective containment or mitigation response to limit the further waves from the COVID-19 pandemic.

## Methods

We extracted publicly available epidemiology data (including total confirmed cases, daily new cases, total deaths, and daily new deaths) from official websites, which included data from the National Health Commission of the People’s Republic of China, and Johns Hopkins University & Medicine Coronavirus Resource Center. To collect the available policy information and sort it out in chronological order, we searched national documents and responses to COVID-19 through various countries’ government web-page, such as media announcements and governmental decrees of these four countries starting from the COVID-19 outbreak.

We assessed the effectiveness of the COVID-19 strategies adopted by these countries through combining the strategies of the four countries with the total confirmed cases, daily new cases, and case-fatality rate.

## Results

### National response in China, Japan, Singapore, and South Korea

#### China

In late December 2019, COVID-19 broke out in Wuhan, China, becoming the first country affected by this virus. On January 23, 2020, China locked down and initiated a large-scale public health intervention. In essence, China’s core prevention and control interventions were the “four early’s” measures and the centralized management of “four categories of patients”. The “four early’s” means that COVID-19 patients should be early detected, reporting, isolated, and treated. And the centralized management of “four categories of patients” is that all confirmed patients were transferred to the hospitals for centralized treatment, suspected patients, febrile patients who might be carriers, and close contacts were sent to designated venues for isolation and medical observation. These policies effectively isolated the source of infection and limited the possibility of alternative transmission routes, while preventing cross-infection. China has curbed the spread of the epidemic across the country through tracing, isolating, and treating COVID-19 patients. On April 8, 2020, Wuhan city was reopened and China had entered into a phase of ongoing prevention and control. Table 1 summarizes China’s major containment strategies.

**Table 1:** The major epidemic prevention and control measures in China

SN	Strategy	Key elements
1	Classification of infectious diseases	On Jan 20, 2020, the COVID-19 included in category B infectious diseases, and adopted prevention and control measures for Category A infectious diseases.
2	Lockdown Wuhan city	On Jan 23, 2020, Chinese authorities adopted unprecedented measures to contain the virus, putting Wuhan city in lockdown. Flights and trains were suspended, and roads were closed. People were told to cannot to leave Wuhan and isolated at home. On April 8, Wuhan reopened.
3	Establishing the command system to prevent the COVID-19	On Jan 25, a leading group was set up by the central government to respond to the COVID-19 outbreak and designated guidance groups to Hubei province and other hard-hit areas.
4	Centralized deployment of materials for epidemic prevention and control	(1) Mobilizing health care workers to support Hubei province and implementing the plan of “Pairing assistance”. As of March 8, there were 346 medical teams with 42,600 medical personnel, supporting Wuhan city and Hubei Province. (2) Establishing Huoshenshan Hospital and Leishenshan Hospital with 1000 and 1600 beds respectively in a short time, and launching 16 Fangcang shelter hospitals, which treated more than 12,000 patients in Wuhan. (3) To ensure the normal operation of the society and implementation of quarantine measures, the government also mobilized more medical supplies and daily necessities to Hubei province.
5	Implementing massive public health measures throughout the entire country	(1) Raising the public health emergency response to the highest level in all localities. (2) Temperature screening point was established in various public places nationwide. (3) Implementing closed or grid management of communities nationwide. Residents were required to take body temperature when they went into communities; imposing extensive public education to residents: home isolation for 14 days after cross-regional travel, wore masks, observe social distancing, reduced public gathering. (4) Taking effective measures to avoid public gatherings and cross infection, such as extended Spring Festival holidays; closed entertainment venues, schools, and workplaces, banned public gatherings, and encouraged people to telecommute. Public service places that need to be open must take body temperature and wear masks.
6	Classifying management of “four categories of personnel”	Since Feb 2, Wuhan has implemented the classified management of “four categories of personnel”(confirmed cases, suspected cases, febrile patients who might be carriers, and close contacts classified management in designated facilities), ensuring that all of these patients were detected, treated and isolated.
7	“Four early’s” measures	(1) On Feb 3, President Xi Jinping said that need to further strengthen prevention and control, and strictly implement the “four early’s” measures of early detection, early reporting, early isolation, and early treatment. (2) He also said that should be saving lives by improving admission and cure rate, and reducing infection and mortality rate.
8	Epidemic prevention and control enter into normal stage	Since April 29, China’s epidemic situation has been sporadic on the whole, with sporadic cases causing clusters in some areas. Imported cases transmission is almost brought under control, which means that China’s epidemic prevention and control entry into a normal stage.

#### Singapore

On Jan 23, 2020, the Ministry of Health (MOH) in Singapore confirmed the first case of COVID-19. Singapore responded rapidly and aggressively to the virus imposing strict border control measures to prevent imported cases. With positive cases increasing on February 4th, the Singapore government instituted regular community prevention and control measures and initiated a hierarchical diagnosis and treatment mechanism. Before April, the effect of epidemic prevention and control was effective, resulting in only two total deaths. However, with the uncontrolled outbreak of COVID-19 in dormitories of migrant workers, the number of cases increased rapidly. Since April 7, Singapore implemented blocking measures and quarantined migrant workers to contain the outbreak. The blocking measures relaxed in three stages after June to gradually restore normal social life. Table 2 summarizes Singapore’s containment strategies to control the pandemic.

**Table 2:** the major epidemic prevention and control measures in Singapore

SN	Strategy	Key elements
1	Escalating border control measures	(1) Since Jan 3, 2020, temperature and health screening of incoming travelers from Wuhan and extended to all travelers since Jan 29, is in place at all ports of entry. (2) Since Feb 1, Singapore imposed entry restrictions on visitors from China; returning residents and long-term pass holders are subject to a 14-days quarantine. (3) Since March 24, prohibiting short-term visitors and cruise ship stops. (4) Since March 27, everyone who enters Singapore without a Stay Home Notice at a designated facility must wear an electronic tracker.
2	Established a Multi-Ministry Task Force	On Jan 23, the Singapore government set up a Multi-Ministry Task Force to provide central coordination for Whole-of-Government handling of the COVID-19 outbreak.
3	Healthcare measures	(1) Launching the National Centre for Infectious Diseases (NCID), a 330-bed purpose-built infectious diseases management facility with integrated clinical, laboratory, and epidemiologic functions, to isolate and treat the confirmed patients. (2) Activating a network of more than 800 Public Health Preparedness Clinics (PHPCs) to enhance management of respiratory infections in the primary care setting and incentivize residents to seek care at these PHPCs. (3) Big Box was transformed into community care facilities to treat and isolate mild patients.
4	Surveillance and containment measures	(1) The MOH of Singapore established suspected cases of COVID-19 criteria and continuously updated them as the global COVID-19 situation evolved. (2) According to the time and distance of contact with the confirmed cases, the contacts were divided into two categories for observation and tracing: asymptomatic close contacts were placed under compulsory quarantine for 14 days, while lower-risk contacts were put on phone surveillance. (3) On March 20, the Singapore government launched the “Trace Together” APP to track close contacts of confirmed cases.
5	Strict community and social measures	(1) Issue a Stay-Home Notice to enforce residents’ isolation at home and cannot go out, breaching Stay-Home Notice will be facing a severe fine. (2) Before April 5, the government implemented regular community prevention and control measures: focusing on public health education; suspending large-scale activities, implementing holidays and home quarantine orders for different groups, and temperature detection. (3) After April 5, the government implemented strict community and social measures such as closed workplaces and schools, and encouraged people to telecommute.
6	Restored normal social life gradually	Normal social life gradually restored in three stages since June: safe reopening, security transition, and security state. Since June 2, Singapore relaxed the blockade measures and enter into a “safe reopening” phase.

#### South Korea

In South Korea, the first confirmed case occurred on Jan 20, 2020, followed by a small number of confirmed cases 1 month later. A large number of confirmed cases emerged related to a religious group called Shincheonji after Feb 20, and the number of confirmed cases increased rapidly. By March 6, the epidemic of South Korea had stabilized and the daily new cases had dropped. In the early stages of the COVID-19 outbreak, the Korean government adopted some measures (massive testing, drive-through screening points, strict social distancing) to contain the spread of the epidemic that ultimately had great results. So the Korean government relaxed restrictions and began to enter a phase of limited control measures that started on May 6th. Table 3 summarizes the containment strategies implemented by South Korea.

**Table 3:** the major epidemic prevention and control measures in South Korea

SN	Strategy	Key elements
1	Activated the National Emergency response system	(1) Jan 27, Raising the public alert level to orange (3 out of 4 levels). (2) Feb 23, Raising the public alert to the highest level.
2	Border control measures	(1) Since Feb 4, 2020, a special entry procedure was introduced for all passengers entering South Korea from China and gradually extended to all global arrivals. (2) Since April 1, all travelers entering Korea are subject to a 14-day quarantine from the day after arrival.
3	Screening and testing measures	(1) On Feb 18, nationwide screening for workers at nursing homes. (2) On Feb 23, launching Drive-through screening centers. (3) On Feb 24, screening all members of Shincheonji religious group.
4	Implementing massive public health measures nationwide	(1) On Feb 17, public relief hospitals in operation. (2) On Feb 22, the Korean government suspended religious activities held indoors or other outdoor activities in densely populated areas, called for citizens to cooperate with epidemic prevention work. (3) On March 2, the reopening time of schools and kindergartens was postponed from March 9 to March 23 in South Korea. (4) Since March 6, the Korean government banned the export of masks, and restricted the purchase of masks, nationwide distribution of public face masks on March 8. (5) On April 11, the government required that people who breached the self-quarantine rule should be worn electronic wristbands.
5	Blockade measures	The South Korean government imposed strict blockades in Daegu city and North Gyeongsang province on Feb 25.
6	Strict social distancing measures	(1) Citizens in South Korea must wear a mask when they take public transportation such as buses and taxis since May 26. (2) On 28 June, the Korean government released the three phases of social distancing measures and their epidemic prevention strategies. (3) The Korean government declared a secondary level of social distance and banned gatherings of 50 people indoors on August 18 due to a new religious cluster infection. (4) Banning gatherings of more than ten people since August 21. (5) Forcing people to wear face masks in the South Korean capital Seoul since August 24.
7	Relaxed epidemic prevention measures	(1) Since April 19, the Korean government softened social distancing measures. (2) On May 6, South Korea began to enter the phase of normal life and epidemic prevention and control.

#### Japan

The first confirmed case of COVID-19 in Japan was reported on Jan 15, 2020. Since Jan 21, the government had issued a level of risk alert to the public and implemented a series of border control measures to prevent the spread of COVID-19. As the rate of transmission increased in February and March, the Ministry of Health, Labour and Welfare issued the “Basic Policy on COVID-19 Countermeasures”, and the prime minister called on the public to conduct “self-restraint”. The prime minister declared a 1-month “state of emergency” order on April 7 and lifted the order nationwide on May 25. The outbreak in Japan continued to rebound in July and August, but the government did not take or advocate any restrictive measures. Table 4 summarizes the major mitigation strategies implemented by Japan.

**Table 4:** the major epidemic prevention and control measures in Japan

SN	Strategy	Key elements
1	Issued risk alert to the public	(1) On Jan 21, issued level 1 risk warning of infectious diseases to the whole of China. (2) On Jan 23, issued level 2 risk warning of infectious diseases to Wuhan, China. (3) On Jan 24, issued level 3 risk warning of infectious diseases to the whole of Hubei province, including Wuhan city, and suspended travel in China’s Hubei province.
2	Border control measures	(1) On 28 January, the Cabinet of Japan decided to designate novel Coronavirus infectious diseases as “designated diseases” based on the Law of infection and “quarantine diseases” based on the Quarantine Law. Infected people are banned from entering Japan. On Feb 1, the decree allowed authorities to require suspected patients to accept quarantines and be hospitalized, and banning travelers from China’s Hubei and Zhejiang provinces. (2) On April 3, Japan tightened border control measures, banning foreigners from 73 countries and regions, including China. In addition, a person who enters Japan from all countries and regions, whether foreigners or Japanese, are required to be quarantined at home for 14 days.
3	The Ministry of Health, Labour, and Welfare launched the “Basic Policies for Novel Coronavirus Disease Control”	(1) The policies suggested citizens wash hands frequently, observe cough etiquette, and avoid public gatherings. It is also suggested that school closure, companies staggered commute. (2) Patients with mild flu-like symptoms should stay at home unless otherwise specified, and seek medical care after consulting the call center or a family doctor if their conditions change. The elderly and those with underlying diseases are encouraged to seek appropriate medical care at the early stage, given their vulnerability to the infection. (3) Establish the surveillance system to grasp the situation of epidemic in Japan, while switching to use of PCR test for the confirmation of diagnosis necessary to treat pneumonia patients who require hospitalization, in communities where the number of patients continues to increase.
4	Prime Minister Calls for “self-restraint”	On Feb 26, the prime minister recommended that self-restraint remain 2 weeks, so the concerts and theaters were suspended or postponed nationwide. On March 10, the requirement expended the time of self-restraint with 10 days.
5	The prime minister called for nationwide school closure	The prime minister called for primary and secondary schools across the country to suspend classes from March 2 to March 20.
6	Declared “state of emergency” order	(1) The prime minister declared a “state of emergency” order and the establishment of the “new lifestyle” that prevents the spread of infection, including avoiding “3 Cs” (closed spaces, crowded places, and close-contact settings) and basic counter-infection measures such as keeping distance, wearing a mask, and washing hands. (2) On May 25, Japan lifted the “state of emergency” order nationwide.

### Results of the prevention and control measures in China

**Fig. 1:**
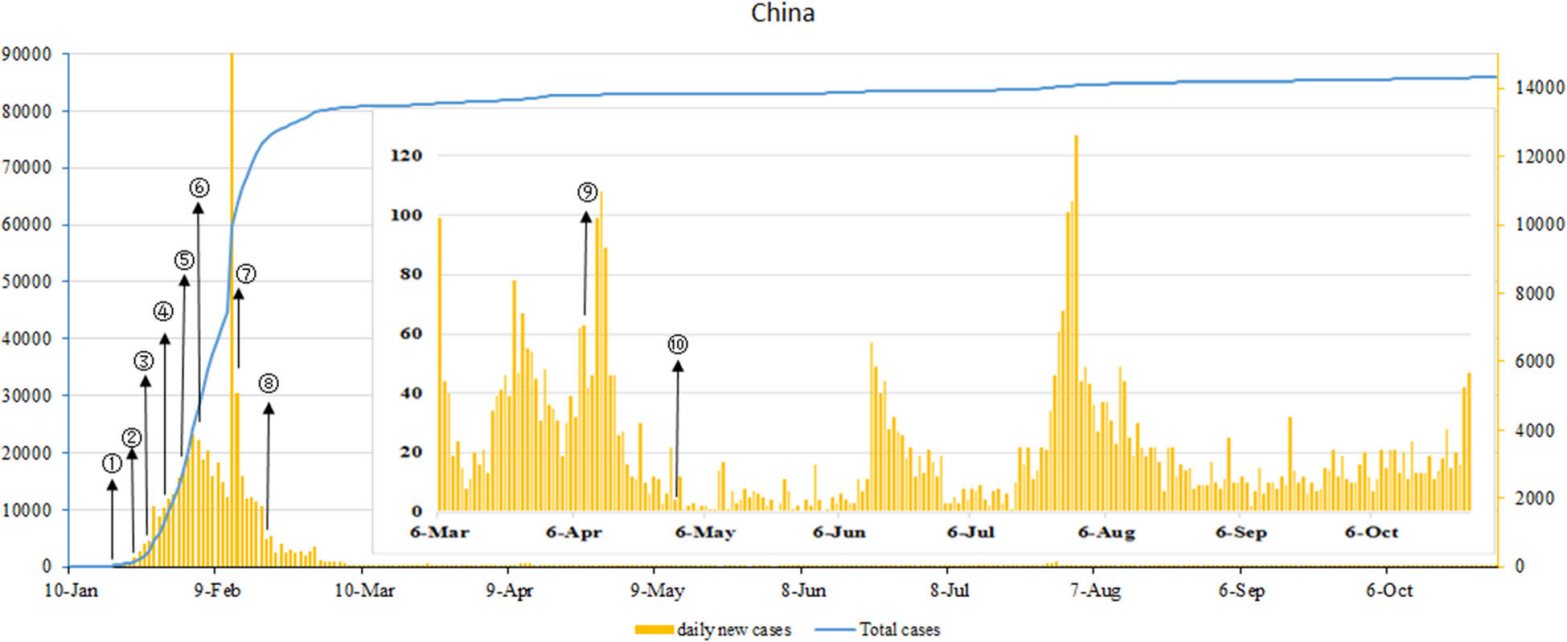
COVID-19 outbreak curve and timeline of implementation of major interventions in China. Note: ① Jan 20, 2020, classification of infectious diseases. ② On Jan 23, Wuhan lockdown. ③ On Jan 25, a leading group was established by the central government to respond to the COVID-19 outbreak. ④ On Jan 29, raising the public health emergency response to the highest level in all localities. ⑤ On Feb 2, classifying management of “four categories of personnel”. ⑥ On Feb 5, makeshift hospitals in operation. ⑦ On Feb 13, pairing assistance. ⑧ On Feb 19, launching massive community screening. ⑨ On April 8, lifted the lockdown of Wuhan city. ⑩ On April 29, China had entered into a phase of ongoing prevention and control

Figure 1 shows the COVID-19 outbreak curve and timeline of implementation of major interventions in China. The confirmed cases of COVID-19 increased exponentially since late January 2020. Especially on Feb 12, the daily new confirmed cases reached a peak with 15,152 cases because Hubei health authorities counted clinically diagnosed cases as confirmed, which resulted in a sharp increase in daily new confirmed cases. Responding to the virus, the Chinese authorities adopted unprecedented containment strategies in mid-January. Such as lockdown epicenter infection areas—Wuhan city on Jan 23. After lockdown, the government classified management of “four categories personnel on Feb 2, built the makeshift hospitals and in operation on Feb 5, paired assistance Wuhan city on Feb 13, and launched massive community screening on Feb 19. These extremely aggressive measures contained a growing epidemic and stopped it in its trajectories in China. The daily new confirmed cases are from thousands per day at the peak down to a couple of dozen since early March. Since April 29, China has entered into an ongoing prevention and control stage and focused on the inbound epidemic.

### Results of the prevention and control measures in Singapore

**Fig. 2:**
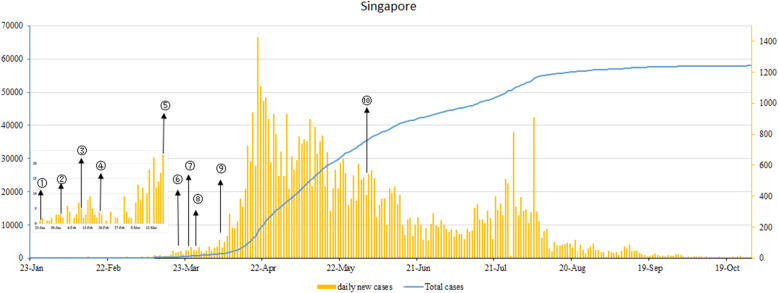
COVID-19 outbreak curve and timeline of implementation of major interventions in Singapore. Note: ① On Jan 23, the first case of COVID-19 was reported and established a Multi-Ministry Task Force. ② Since Feb 1, Singapore imposed entry restrictions on visitors from China; returning residents and long-term pass holders are subject to a 14-days quarantine. ③ On Feb 10, Activating a network of more than 800 (PHPCs). ④ On Feb 18, issued isolation decree. ⑤ Since March 17, inbound travelers who have symptoms such as fever and cough must take throat swabs. ⑥ On March 30, the government of Singapore launched the “Trace Together” APP. ⑦ Since March 24, prohibiting short-term visitors and cruise ship stops. ⑧ Since March 27, everyone who enters Singapore without a Stay Home Notice at a designated facility must wear an electronic tracker. ⑨ Since April 5, the government implemented strict community and social measures and encouraged people to telecommute. ⑩ Since June 1, normal social life has gradually been restored in three stages

Figure 2 shows the COVID-19 outbreak curve and timeline of implementation of major interventions in Singapore. Singapore responded rapidly and aggressively to the COVID-19 outbreak and implemented strict containment measures in the early phase. Such as the authorities established a Multi-Ministry Task Force on Jan 23, 2020, returning residents or long-term pass holders are subjected to a 14-days quarantine since Feb 1, activated a network of more than 800 PHPCs on Feb 10, and launched the “Trace Together” APP on March 30. So the daily new confirmed cases remain at a low level. But since April 2020, the daily new confirmed cases increased sharply due to the migrant worker dormitory outbreaks. In response to the new cluster infections, Singapore authorities quickly tightened entry restrictions, closed nonessential business, and reinforced strict order on social distancing since April 5. So since August, the daily new confirmed cases have decreased.

### Results of the prevention and control measures in South Korea

**Fig. 3:**
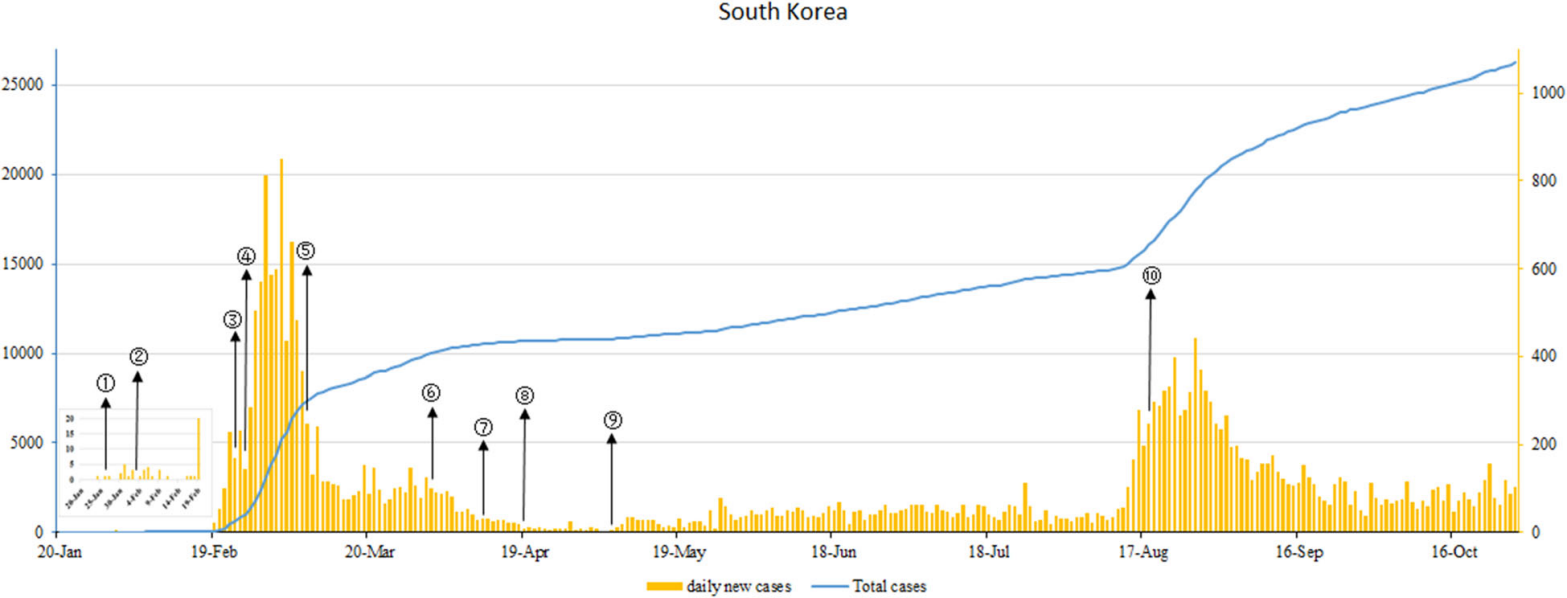
COVID-19 outbreak curve and timeline of implementation of major interventions in South Korea. Note: ① Jan 27, raising the public alert level to orange (3 out of 4 levels). ② Since Feb 4, a special entry procedure was introduced for all passengers entering South Korea from China and gradually extended to all global arrivals. ③ Feb 23, raising the public alert to the highest level and launching Drive-through screening centers. ④ On Feb 25, the Korean government imposed strict blockades in Daegu city and North Gyeongsang province. ⑤ On March 8, the nationwide distribution of public face masks. ⑥ Since April 1, all travelers entering South Korea are subject to a 14-day quarantine from the day after arrival. ⑦ On April 11, the government required that people who breached the self-quarantine rule should be worn electronic wristbands. ⑧ On April 19, softening social distancing measures. ⑨ On May 6, South Korea began to enter the phase of normal life and epidemic prevention and control. ⑩ On August 18, the Korean government declared a secondary level of social distance and banned gatherings of 50 people indoors

Figure 3 shows the COVID-19 outbreak curve and timeline of implementation of major interventions in South Korea. In the early stage of the COVID-19 outbreak, the Korean government raised the public alert level to orange (3 out of 4 levels) on Jan 27, 2020. A Special Entry Procedure was introduced for all passengers entering South Korea from China and gradually extended to all global arrivals since Feb 4, and raised the public alert to the highest level and launched Drive-through screening centers on Feb 23. These containment measures had led to the daily new confirmed cases of South Korea were a single-digit increase. However, the epidemic curve has risen rapidly since Feb 19, 2020, this was linked to the new cluster infection of Shincheonji (a Korean religious movement from Daegu). Responding to this outbreak, the South Korean government imposed strict blockades in Daegu city and North Gyeongsang province on Feb 25, distributed public face masks nationwide on March 8. All travelers entering South Korea are subject to a 14-day quarantine from the day after arrival since April 1 and required that people who breached the self-quarantine rule should be worn electronic wristbands on April 11. So the daily new confirmed cases have dropped. But since mid-August, the daily new confirmed cases appeared a rapid increase due to an again cluster infection of the church.

### Results of the prevention and control measures in Japan

**Fig. 4:**
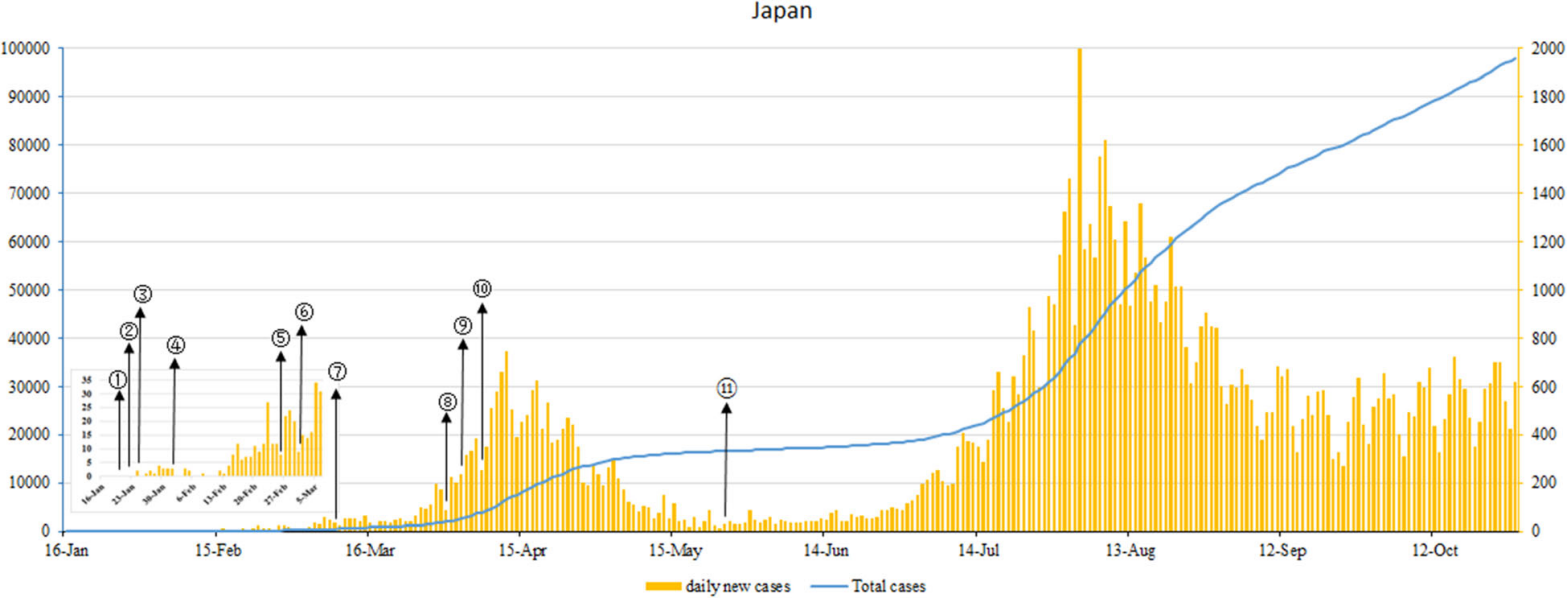
COVID-19 outbreak curve and timeline of implementation of major interventions in Japan. Note: ① On Jan 21, issued level 1 risk warning of infectious diseases to the whole of China. ② On Jan 23, issued level 2 risk warning of infectious diseases to Wuhan, China. ③ On Jan 24, issued level 3 risk warning of infectious diseases to the whole of Hubei province, including Wuhan city, and suspended travel in China’s Hubei province.④ On Feb 1, the decree allowed authorities to require suspected patients to accept quarantines and be hospitalized, and banning travelers from China’s Hubei and Zhejiang provinces. ⑤ On Feb 26, the prime minister recommended that self-restraint remains for 2 weeks, so the concerts and theaters are suspended or postponed nationwide. ⑥ On March 2, nationwide school closure. ⑦ On March 9, Japanese who entered from China and South Korea should be isolated at designated places. ⑧ On March 31, Japan prohibited visitors entering from 49 countries or regions (such as United States, England, China, South Korea, and so on), and also advised their citizens not to travel to these countries and regions. ⑨ On April 3, Japan tightened border control measures, banning foreigners from 73 countries and regions, including China. ⑩ On April 7, the prime minister declared a “state of emergency” order and the establishment of the “new lifestyle” that prevents the spread of infection, including avoiding “3Cs” (closed spaces, crowded places, and close-contact settings). ⑪ On May 25, Japan lifted the “state of emergency” order nationwide

Figure 4 shows the COVID-19 outbreak curve and timeline of implementation of major interventions in Japan. At the initial stage of the COVID-19 epidemic, the Japanese Ministry of Foreign Affairs gradually raised the risk alert level for the epidemic in January. In February, the decree allow suspected patients to accept quarantines and be hospitalized, and the prime minister recommended that self-restraint remains for 2 weeks. In March, nationwide school closure and the authorities issued strict border control measures. So the daily new confirmed cases increased slowly. But since April 2020, the daily new confirmed cases have increased rapidly. In response to the increase of the daily confirmed cases, Japan tightened border control measures, banning foreigners from 73 countries and regions, including China on April 3. Meanwhile, the Japanese prime minister announced a state of emergency to contain the outbreak on April 7, and in late-May, the epidemic curve dropped. So on May 25, the Japanese government lifted a state of emergency. However, the epidemic rebound since July and the daily new confirmed cases increased more sharply than before.

### The total confirmed cases, total deaths, and mortality of four countries

**Fig. 5:**
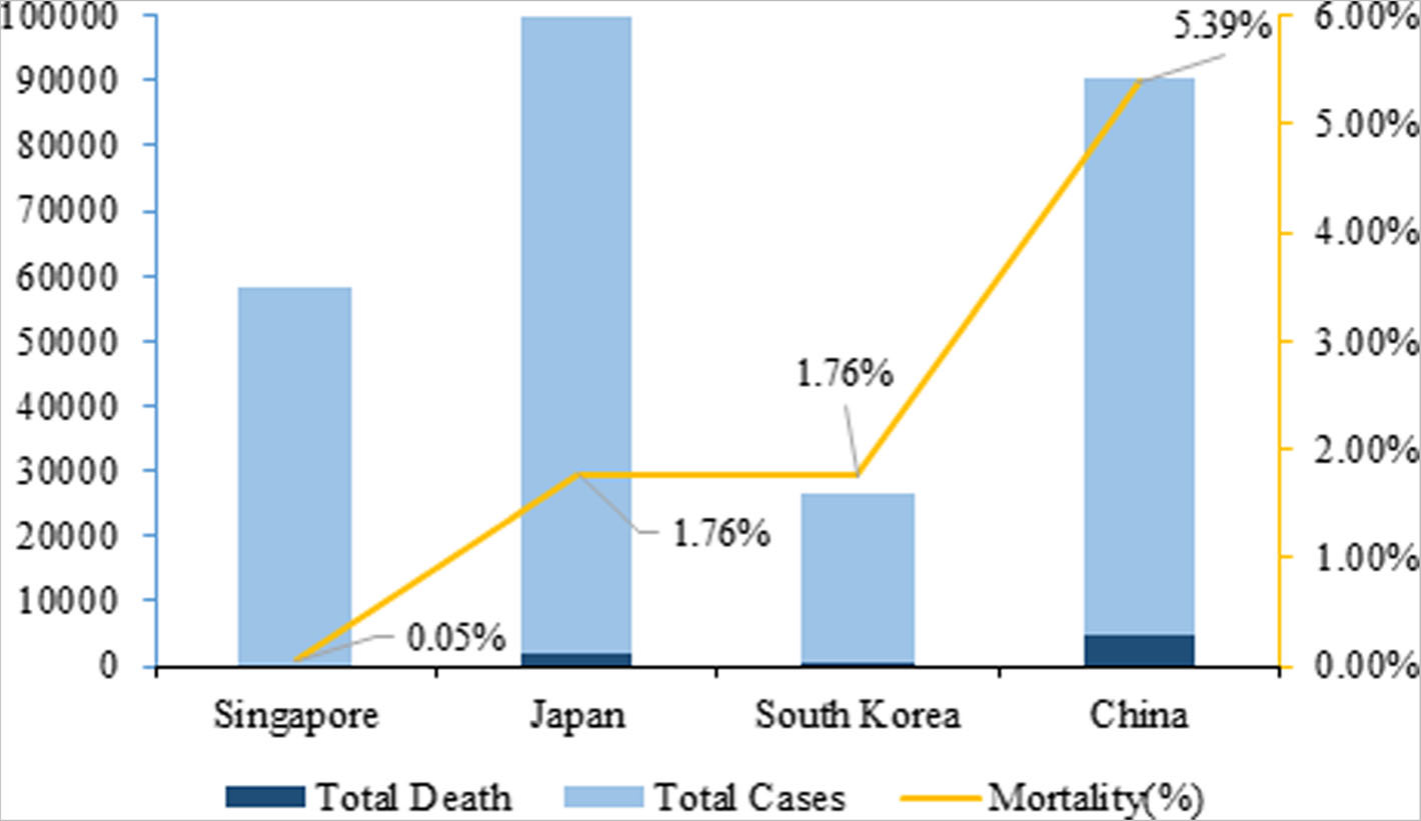
Total cases, total death and case-fatality rates in four countries (as of October 28, 2020)

Figure 5 shows the total confirmed cases, total deaths, and mortality of the four countries. As of October 28, the total confirmed cases of these four countries remain under 100,000 with Japan having the highest. In four countries, China’s case-fatality is highest at 5.39%, Singapore’s case-fatality is lowest at 0.05%, and the case-fatality of South Korea and Japan are the same with 1.76%.

## Discussion

Our study presents the COVID-19 situations and evaluated the efficiency of the prevention and control measures in China, Japan, Singapore, and South Korea. We found that the epidemic of China, Singapore, and South Korea was well control through implementing a containment strategy. Whereas, Japan has adopted a mitigation strategy, averting a rapid increase of COVID-19 cases. But mitigation strategy is unlikely to block the virus transmission, so the second wave epidemic of Japan seems to increase more sharply than before. Fortunately, these four countries have kept the new infection numbers at a low level, whether China, Singapore, and South Korea had imposed a containment strategy or Japan had utilized a mitigation strategy.

### Containment strategy

Whether in China and South Korea that the epidemic was serious in the early stage, or in Singapore that the imported epidemic was predominant, the epidemic has been better controlled by actively adopting rigorous containment strategies, even interrupting the local transmission of the virus. Except for China, which had a high rate of case-fatality (5.39%) in its early phase due to the crowding out of medical resources, both Singapore and South Korea had relatively low case-fatality with 0.05 and 1.76% respectively. But some policy disparities existed among these three countries.

China, which was affected by the novel coronavirus from the early stages, instituted unprecedented containment measures—locking down Wuhan city to block the COVID-19 transmission. Subsequently, varying degrees of intra-area and inter-area transportation restrictions were applied across the country, from big cities to small villages, for at least 1 month [5]. After locking down infectious areas, the Chinese government proactive in finding and managing “four categories of personnel” — confirmed cases, presumptive cases, fever cases, and close contacts. Furthermore, to control the source of infection and prevent the virus spreading to the wider regions, the authorities do the utmost efforts to quarantine or treat these patients through increasing designated hospitals or facilities, establishing makeshift hospitals, and mobilizing health care workers to assistance Hubei province [6]. Meanwhile, between May 14 and June 1, 2020, a mass citywide nucleic acid screening of the SARS-CoV-2 infection screening program was setting in the post-lockdown Wuhan, recruiting nearly 10 million people [7]. In this way, these containment measures could as early as possible to control the source of infection and keep a new infection at a low level.

Singapore adopted a containment strategy of flattening the curve, which was minimizing the spread of the virus through early detection, early isolation, and early treatment, avoiding the crowding out of health resources and the collapse of the system [8]. The most characteristic of Singapore’s epidemic control is its strong surveillance system and public health system. To detect cases accurately and effectively, the “Trace Together” APP was launched that allowed authorities to identify individuals who have been in close contact or exposed to infected patients, and trace their movements on March 20, 2020 [9]. Also, the Singapore government activated a network of more than 800 Public Health Preparedness Clinics (PHPCs) to enhance the management of respiratory infections in the primary care setting, with subsidies extended to Singapore residents to incentivize them to seek care at these PHPCs [5,9]. Furthermore, most of the COVID-19 patients can also isolated and treated at the National Centre for Infectious Diseases (NCID), a 330-bed purpose-built infectious diseases management facility. Singapore has also implemented other measures to control the spread of COVID-19. To prevent imported cases causing local transmission, strict border measures included temperature and health screening, entry restrictions, and 14-days quarantine orders were implemented [10]. To reduce community transmission, Singapore has implemented strict community and social measures due to rapid increase, through the closure of schools, workplaces, and shops since April 5.

South Korea, a country without extremely regional lockdowns, has greatly slowed the initial epidemic. Korea’s containment response mainly its thorough quarantine and contact tracing system. The government made an effort to identify undiagnosed patients immediately after confirming diagnosed patients by tracking their route and finding the infection source. Once someone was identified as close contact, he or she was immediately required to self-isolation, and their health status was constantly monitored by the government. Thereby, they can be diagnosed promptly and received treatments in a timely manner when they develop relevant symptoms, thus lowering morbidity and case-fatality. Furthermore, to sample collection coupled with fast and aggressive testing, drive-through and walk-through screening stations were introduced for allowed early detection of confirmed cases in communities [11]. With these stations in place, even asymptomatic patients did not miss diagnosed, making Korea’s COVID-19 statistics more reliable [12]. In this way, Korea has substantially slowed down the spread of the virus. On October 28, 2020, South Korea reported only 113 daily new cases, decreasing from 851 cases at its first peak on March 3, 2020, and 441 at its second peak on August 27, 2020 [13].

### Mitigation strategy

Japan is a typical representative country that has implemented mitigation strategies to reduce the spread of virus transmission. In the initial stage of the epidemic, the Japanese government clearly stated that focus on the treatment of severe cases, patients with very mild illness were generally advised to stay at home, and asymptomatic people were discouraged from being tested for the new coronavirus [14]. Furthermore, Japan prioritized policy on restricting large-scale clusters, declared a one-month “state of emergency” order on April 7, 2020, and allowed the government to impose social distancing measures [9]. But because its intervention strategy is unable to prevent the continued spread of the epidemic, the second wave of the outbreak since July has appeared to be somewhat more severe than the first, with the number of confirmed cases increasing more rapidly than before. However, thanks to the self-discipline and high health literacy of the Japanese people, Japan has not experienced the rapid increase in cases as in European countries, and has become one of the countries with exceptional performance in the development of the epidemic among countries implementing mitigation strategies.

## Conclusion

This study found that China, Japan, Singapore, and South Korea accessed the situation within their own borders and implemented different intervention strategies to curb the spread of COVID-19 and maintain lower rates of case-fatality. China, Singapore, and South Korea adopted the containment strategy, while Japan adopted the mitigation strategy. Although Japan’s case-fatality maintained at a low level, daily new cases increased faster than the other three countries. This result indicated that a mitigation strategy could be inferior to a containment strategy. Countries could choose the appropriate strategy for response to the COVID-19 pandemic based on their own situation.

## Data Availability

All data generated or analyzed during this study are included in this published article.
